# A One-Step Electropolymerized Biomimetic Polypyrrole Membrane-Based Electrochemical Sensor for Selective Detection of Valproate

**DOI:** 10.3389/fbioe.2022.851692

**Published:** 2022-02-15

**Authors:** Yuyang Yuan, Tianyu Li, Zhichao Ye, Yuyao Feng, Zhe Chen, Yusen Wang, Yiqiao Sun, Haoyu Wu, Zhaodong Yang, Yifan Wang, Yiran Zhang, Liquan Huang, Bo Liang

**Affiliations:** ^1^ Biosensor National Special Laboratory, College of Biomedical Engineering and Instrument Science, Zhejiang University, Hangzhou, China; ^2^ College of Life Sciences, Zhejiang University, Hangzhou, China; ^3^ School of Medicine, Zhejiang University, Hangzhou, China; ^4^ School of Biological Sciences, The University of Hong Kong, Hong Kong, Hong Kong SAR, China; ^5^ College of Agriculture and Biotechnology, Zhejiang University, Hangzhou, China; ^6^ College of Control Science and Engineering, Zhejiang University, Hangzhou, China; ^7^ Department of Biochemistry, Yong Loo Lin School of Medicine, National University of Singapore, Singapore, Singapore; ^8^ Department of Chemistry, Zhejiang University, Hangzhou, China; ^9^ Shanghai Institute for Advanced Study of Zhejiang University, Shanghai, China

**Keywords:** valproate, bipolar disorder, electrochemical sensor, molecularly imprinted polymer, drug detection

## Abstract

Bipolar disorder is a chronic mental disease with a heavy social and economic burden that causes extreme mood swings in patients. Valproate is a first-line drug for bipolar disorder patients to stabilize their daily mood. However, an excessive amount of valproate in the blood could induce severe adverse effects, which necessitates the monitoring of blood valproate levels for patients. Here, we developed an innovative electrochemical sensor for selective and simple detection of valproate based on a molecularly imprinted polymer membrane *via* one-step electropolymerization. Gold nanoparticles were electrochemically modified to the screen-printed electrode under the selective membrane to enhance its conductivity and stability. The successfully fabricated biosensor was characterized by scanning electron microscopy, cyclic voltammetry, and differential pulse voltammetry methods. The binding of the target molecules to the valproate-customized biomimetic polypyrrole membrane blocks cavities in the membrane and alters its electric properties, which can be detected as a decrease in the peak current by differential pulse voltammetry method. The peak current change presents a great log-linear response to the valproate concentration around the therapeutic window. The limit of detection of this method was 17.48 μM (LOD, S/N = 3) and the sensitivity was 31.86 μM μA^−1^. Furthermore, the biosensors exhibited both satisfying specificity with the interference of other psychological pharmaceutical drugs and uniformity among sensors, indicating their potential and reliability in translational application. This simple and reliable method of sensing valproate molecules primarily provides an exceptional solution to valproate point-of-care testing in clinical practice.

## Introduction

Bipolar disorder (BD) is a recurrent chronic disorder featured with fluctuations in mood state and energy, which affects more than 1% of the world’s population. Patients with BD experience mania and depression alternately, which usually compromise their psychosocial functioning (Grande et al., 2016). For BD management, medication including valproate (VPA) is primarily recommended (Bond et al., 2010; Grande et al., 2016).

Since its first introduction in 1964, VPA has been widely used clinically as a broad-spectrum antiepileptic drug and particularly as a typical mood stabilizer for BD treatment. It is one of the four drugs approved by FDA for BD acute mania episode management probably by promoting the synthesis of gamma-aminobutyric acid, an inhibitory neurotransmitter that reduces neuronal excitability (Löscher, 2002; Li et al., 2002; Bialer, 2012; Grande et al., 2016). While VPA has been widely prescribed and proven effective for BD episode control, it has a narrow therapeutic window, and its overdosage was reported to trigger severe side effects such as liver and kidney impairment and nervous system disorders (Nanau and Neuman, 2013). It is strongly recommended that blood levels of such drugs with limited therapeutic concentration should undergo therapeutic drug monitoring (TDM) due to the individual heterogeneity in pharmacokinetics (Hiemke et al., 2018). Therefore, patients who take VPA are supposed to be regularly followed up and even be hospitalized for TDM followed by dose adjustment in order to achieve satisfying therapeutic effects, which adds a heavy economic burden and workload to patients and hospitals. Apart from the canonical applications mentioned earlier, VPA was also reported as a potent histone deacetylase inhibitor, and early clinical studies have revealed its promising potency in cancer therapy (Cincarova et al., 2013), indicating its broader application prospect and the need for rapid determination of its plasma concentration.

Currently, clinical VPA-TDM is basically carried out by HPLC (Eap and Baumann, 1996; Hiemke et al., 2018). Despite its high accuracy, HPLC has drawbacks such as complicated procedures, large time consumption, and bulky equipment. Point-of-care testing (POCT) is just to solve these problems. Contrary to the traditional laboratory test, POCT is featured as fast detection and portable device, and brings analysis methods closer to patients in terms of both time and space (Luppa et al., 2011). Among various analysis methodologies, the electrochemical method has been widely adopted in POCT for low cost, easy operation, size minimization, and real-time response (Islam et al., 2020). With an electrochemical POCT device, doctors and even patients themselves can easily determine the VPA blood concentration from bedside within a few minutes, saving the time of sending the sample to the medical laboratory and waiting for the results, which usually takes several hours to days. However, few previous studies detected VPA based on electrochemical methods, owing to the lack of recognizable functional groups as well as the reactivity inertness of VPA on the electrode. Only a solid VPA^−^ ion-selective sensor based on VPA-dopped conducting polypyrrole film was developed, yet it responded to other anions as well. As blood carries various anions, the lack of selectivity hindered its application to real samples (Sabah et al., 2006).

Molecularly imprinted polymer (MIP) is a kind of artificial receptor with tailor-made recognition sites (Chen et al., 2011). It is synthesized by the polymerization of functional monomer (serves as the backbone of the membrane) containing templates (target analytes). After template molecules are removed from the polymer, cavities complementing the template molecules in shape, size, and functional groups are exposed, which have high affinity with the template molecules. Given the advantages of high selectivity, physical robustness, simple fabrication, and low cost (Ahmad et al., 2019), the MIP has been widely applied in solid-phase extraction (Arabi et al., 2020), small-molecule determination (Parlak et al., 2018), and protein and cell recognition (Wang et al., 2016). MIP-based electrochemical sensors have been applied in the determination of some neurological drugs, like L-dopa (Lin et al., 2015), but none of them focused on VPA.

Screen-printed electrodes (SPEs) have been widely used in electrochemical biosensors. SPEs are cheaper, easier, and faster than the traditional electrodes for mass production, and reduce the required sample volumes, which contributes to the miniaturization of the device. SPEs can be easily modified by various materials, customized for different sensing purposes (Taleat et al., 2014; Beitollahi et al., 2020; Mincu et al., 2020).

**FIGURE 1:**
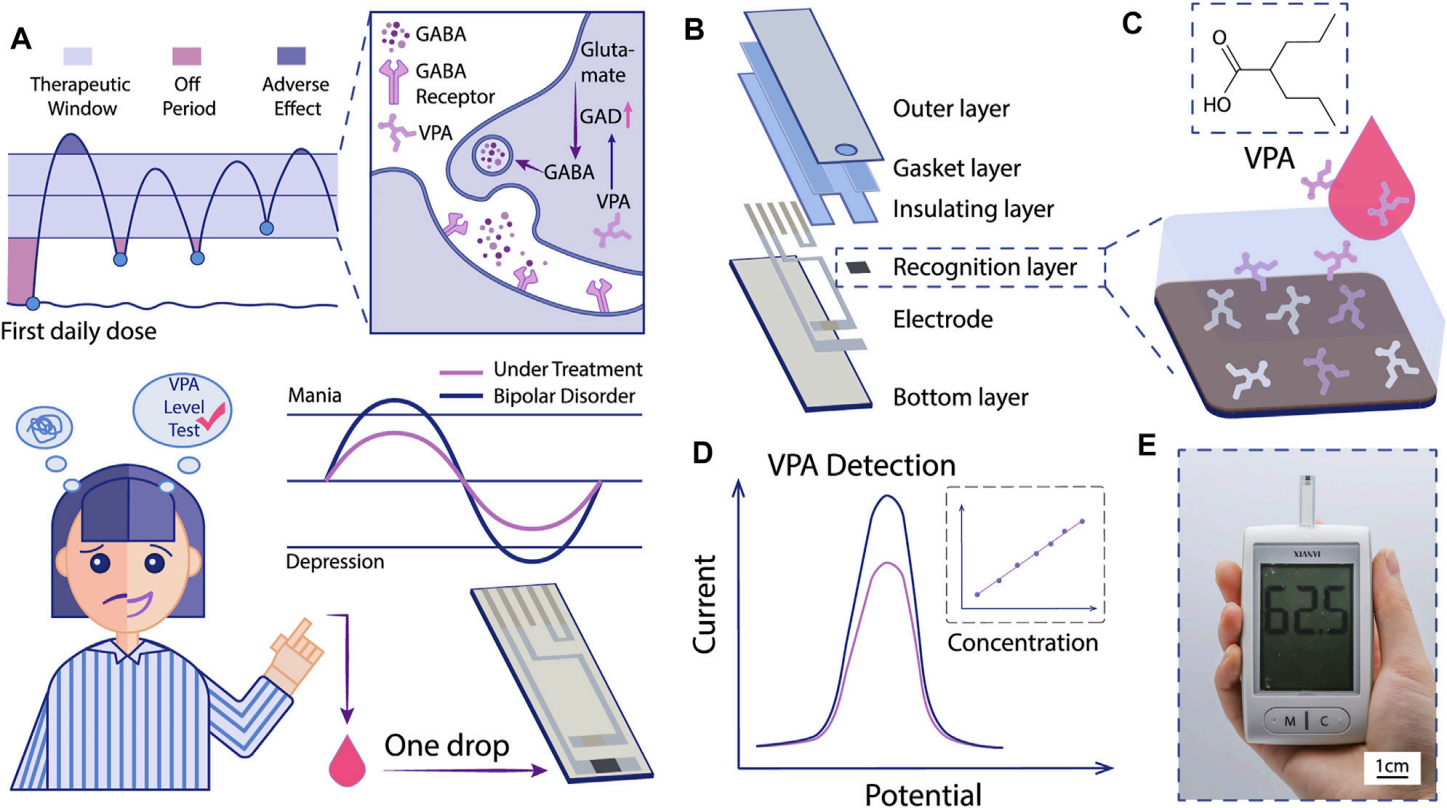
Schematic illustration of the MIP-based electrochemical sensor for VPA-POCT. **(A)** The mechanism of VPA for BD treatment and the hopeful application of MIP-based electrochemical sensor for VPA-POCT. GAD: glutamic acid decarboxylase. GABA: gamma-aminobutyric acid. **(B)** The structure of the sensor. Specific binding of VPA molecules to MIP **(C)** induced a signal change **(D)**. **(E)** The recorded signals can be interpreted into VPA concentrations with a portable device.

Herein, we developed a novel selective electrochemical biosensor for fast and reliable detection of VPA based on a one-step electropolymerized biomimetic ppy membrane which functioned as the recognition layer (Figures 1A,B). Polypyrrole (ppy) is a typical conductive polymer with controlled conductivity and high stability (Sharma et al., 2012). In the present study, pyrrole was used as the functional monomer to fabricate the MIP by electropolymerization. The ppy-MIP was modified to the SPE dotted with gold nanoparticles (AuNPs), where AuNPs were introduced to improve the conductivity, to get ppy@AuNPs-MIP senor. The designed sensor exhibited great electrochemical performance in cyclic voltammetry (CV) and differential pulse voltammetry (DPV) methods. Selective binding of the VPA molecule to the MIP led to a signal change in DPV, which was proven to bear a high relevance to the VPA concentration and reflect the VPA concentration in test samples (Figures 1C,D). The sensor stood the interference test and showed good specificity for VPA. Moreover, since the entire procedure was highly controllable, the consistency of detection performance among sensors was as expected. Based on these results, our sensor can hopefully be integrated into a miniaturized portable device, which is promising to be an alternative choice in VPA-TDM, providing convenience for both patients and medical organizations (Figure 1E).

## Materials and Methods

### Chemicals and Materials

Chloroauric acid (HAuCl_4_), pyrrole, lithium perchlorate (LiClO_4_), sodium valproate (VPA), potassium hexacyanoferrate (III) (K_3_[Fe(CN)_6_]), potassium hexacyanoferrate (II) trihydrate (K_4_[Fe(CN)_6_]·3H_2_O), potassium chloride (KCl), di-potassium hydrogen phosphate trihydrate (K₂HPO₄·3H₂O), and quetiapine were purchased from Aladdin Ltd. (Shanghai, China). Lamotrigine, aripiprazole, ziprasidone, and carbamazepine were obtained from RHAWM Ltd. (Shanghai, China).

All reagents in this work were used without further purification. All solutions were prepared in deionized (DI) water (resistivity of 18 MΩ) purified in the water purification system SMART-N (Heal Force, China).

### Apparatus and Characterizations

Electrochemical experiments such as cyclic voltammetry (CV) and differential pulse voltammetry (DPV) were performed on an AUTOLAB M204 workstation (Metrohm, Switzerland) with designed screen-printed electrodes (SPEs) (Shandong Industrial Technology Research Institute of Zhejiang University). Scanning electron microscope (SEM) images were obtained on a field emission scanning electron microscope (Nova NanoSEM 450, FEI, Eindhoven, Netherlands) with an acceleration voltage of 10 kV to characterize the modified electrode surface morphology.

### Preparation of ppy@AuNPs-MIP

SPE was washed with deionized water and dried in nitrogen (N_2_) gas at room temperature before experiments. Then the cleaned SPE was immersed into 10 mM HAuCl_4_/5 mM HCl, and a constant voltage of 0 V was applied for 100 s according to the literature (Zhu et al., 2020), obtaining the AuNP-modified carbon electrode. Afterward, the SPE was immersed in the polymerization solution, which was de-aerated by bubbling N_2_ gas. The MIP electrode was prepared by electrodeposition of pyrrole onto the surface of the SPE using cyclic voltammetry in the potential range between −0.6 and 0.8 V during seven cycles (scan rate 50 mV s^−1^) in the aqueous solution of 0.025 M pyrrole, 0.01 M VPA, and 0.1 M LiClO_4_ (supporting electrolyte). After the electropolymerization process, the entrapped template, VPA, was removed by applying a constant potential of +1.3 V (Yu et al., 2019) for 20 min in 0.2 M K₂HPO₄ solution, leaving vacancies complementary in shape and functionality to the original template VPA. The fabricated electrode was labeled as ppy@AuNPs-MIP-modified electrode.

A control electrode [non-imprinted polymer (NIP) modified electrode] was fabricated with the same procedure, but the polymerization solution was made up of 0.025 M pyrrole and 0.1 M LiClO_4_ (i.e., without VPA).

### Electroanalytical Measurements

SPE with different modifications was used as the working electrode, with platinum as the counter electrode and Ag/AgCl as the reference electrode. All the electrodes were rinsed with DI water and dried before each measurement. Cyclic voltammetry (CV) experiments were carried out in 0.01 M [Fe(CN)_6_]^3−/4−^and 0.1 M KCl at a scanning rate of 50 mV s^−1^ in the potential range of −0.1–0.5 V. Differential pulse voltammetry (DPV) was carried out in a solution containing 0.01 M [Fe(CN)_6_]^3−/4−^ and 0.1 M KCl between −0.05 and 0.5 V at a scan rate of 100 mV s^−1^. All the measurements of MIP and NIP in this study were performed in triplicates.

## Results and Discussion

### Molecularly Imprinting Electropolymerization

**FIGURE 2:**
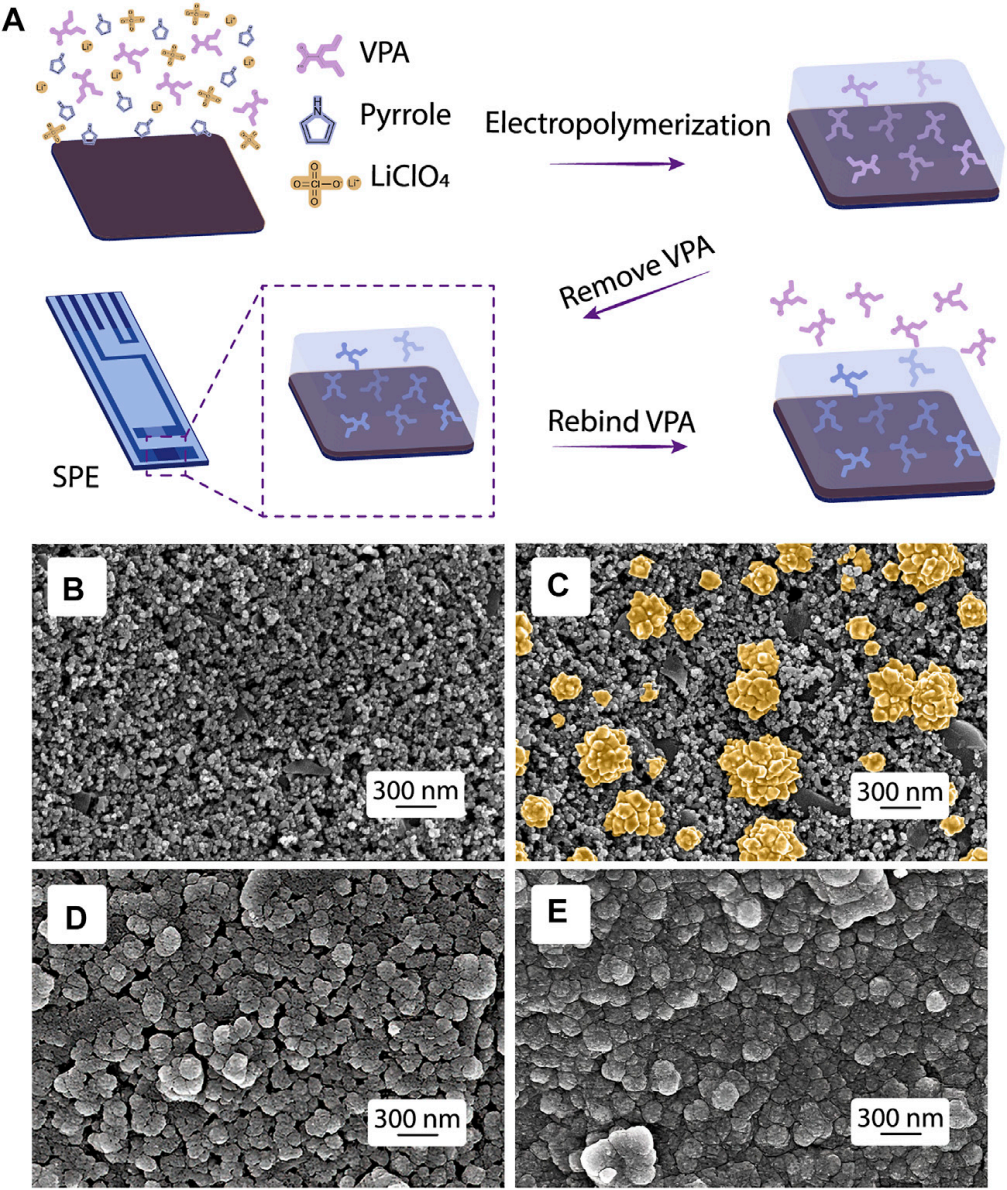
MIP fabrication and morphology characterization. **(A)** Scheme of the MIP synthesis. SEM images of **(B)** bare SPE, SPEs modified with **(C)** AuNPs, **(D)** ppy@AuNPs-MIP, and **(E)** ppy@AuNPs-NIP.

**FIGURE 3:**
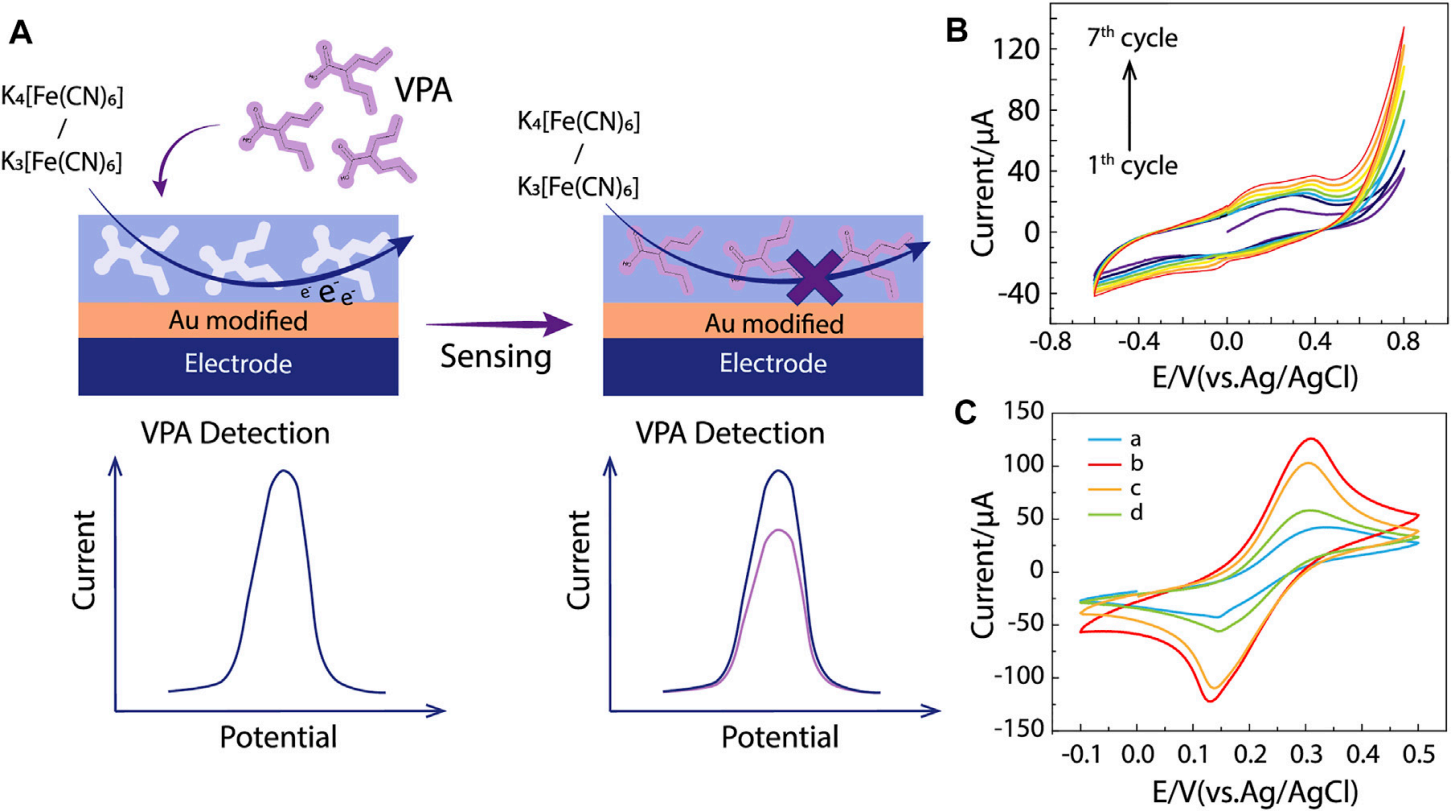
MIP/gate effect and electrochemical characterization. **(A)** The major principle of MIP/gate effect. **(B)** Cyclic voltammogram of electropolymerization in a solution containing 0.025 M pyrrole, 0.01 M VPA, and 0.1 M LiClO_4_, from −0.6 to 0.8 V, at scan rate 50 mV s^−1^ (vs. Ag/AgCl). **(C)** CV responses of 0.01 M [Fe(CN)_6_]^3−/4−^ solution containing 0.1 M KCl at **(a)** bare SPE, SPEs modified with **(b)** ppy@AuNPs-MIP, **(c)** ppy@AuNPs-MIP incubated in VPA solution, and **(d)** ppy@AuNPs-NIP.

The synthesis of ppy@AuNPs-MIP is illustrated in Figure 2A. Electrodeposition of the AuNPs on carbon SPE increased the conductivity of the electrode to improve the sensibility of the sensor. Based on the chemical structure, a relatively strong hydrogen bond interaction could be formed between VPA (the oxygen atom in the carboxyl group) and pyrrole (the hydrogen atom in the N-H group). Meanwhile, VPA (pK_a_ = 4.6), existing as an anion in electropolymerization solution, was electrostatically attracted to the positively charged ppy skeleton. Therefore, during the electropolymerization process of pyrrole, the VPA molecules were captured and embedded in the ppy matrix to form a recognition site complementary to the shape, size, and function of the VPA molecule. With the polymerization process, the CV-response current increased as the number of scanning cycles increased (Figure 3B), indicating the constant addition of monomers to the growing conductive polymer. The exertion of constant potential +1.3 V overoxidized the ppy membrane and deprived it of electropositivity. Therefore, the embedded VPA molecules were extracted and recognition sites became exposed, which were ready for the recognition and rebinding of VPA molecules in the analyzing solution. The overoxidation of ppy membrane provided it with a larger surface area, facilitating a more sensitive detection. Also, it introduced various functional groups such as carbonyl and carboxyl to the membrane, thus improving the sensor’s selectivity (Shiigi et al., 2003; Ahmad et al., 2019).

### Morphological Characterization of the ppy@AuNPs-MIP and ppy@AuNP-NIP

The morphology of the bare SPE and SPEs modified by AuNPs, ppy@AuNPs-MIP, and ppy@AuNPs-NIP was characterized by SEM (Figures 2B–E). At a 300-nm scale, flower-like AuNPs were uniformly distributed on the electrode surface, which provided a larger reaction area and more catalytic sites for electrochemical reactions. After electropolymerization, the SPEs were covered with a polypyrrole membrane, whose granules were larger than granules in bare SPE. As for ppy@AuNPs-MIP and ppy@AuNPs-NIP, the gap space in MIP was much larger than that in NIP. It suggested that the VPA molecules, which occupied the recognition sites earlier, were successfully removed. On the other hand, the VPA molecule was absent in NIP fabrication, so there were no corresponding recognition sites.

### Electrochemical Characterization

Since VPA can hardly be electrochemically oxidized or reduced, it is difficult to detect it with electrochemical methods directly. Utilizing electroactive probes [Fe(CN)_6_]^3−/4−^ is a favorable solution taking advantage of the MIP/gate effect. The effect refers to the change of reduction peak current (ΔI) before and after the analytes bind to the MIP. Specifically, [Fe(CN)_6_]^3−/4−^ in solutions has access to the surface of the electrode through vacant recognition sites and induces electrical signals, yet once VPA occupies the recognition site, it inhibits the electron transformation of [Fe(CN)_6_]^3-/4-^ on the electrode, reducing its reduction peak current (Figure 3A). The amount of change reflects the concentration of VPA in the solution. Additionally, this indirect strategy has a higher sensitivity than electrochemical catalysis, which contributes to the better analytical performance of our sensor (Liu et al., 2017).

As shown in Figure 3C, the CV method was applied to SPEs modified by AuNPs, ppy@AuNPs-NIP, and ppy@AuNPs-MIP (before and after incubation in VPA solution for 8 min). Curve A showed a reduction peak around +0.3 V, which belonged to [Fe(CN)_6_]^3−/4−^. When the bare SPE was modified by ppy@AuNPs-MIP, the peak current intensity increased obviously (Figure 3C, curve b). This phenomenon was attributed to the porosity of the MIP. The hollow recognition sites in MIP expanded the surface area, promoting the electron transformation of [Fe(CN)_6_]^3−/4−^. After being incubated in VPA solution, the recognition sites were occupied and the electron transfer was restricted, so the peak current dropped significantly (Figure 3C, curve c). On the contrary, the SPE modified with ppy@AuNPs-NIP exhibited a lower peak current intensity (Figure 3C, curve d), owing to the absence of available recognition sites.

### Determination of VPA

**FIGURE 4:**
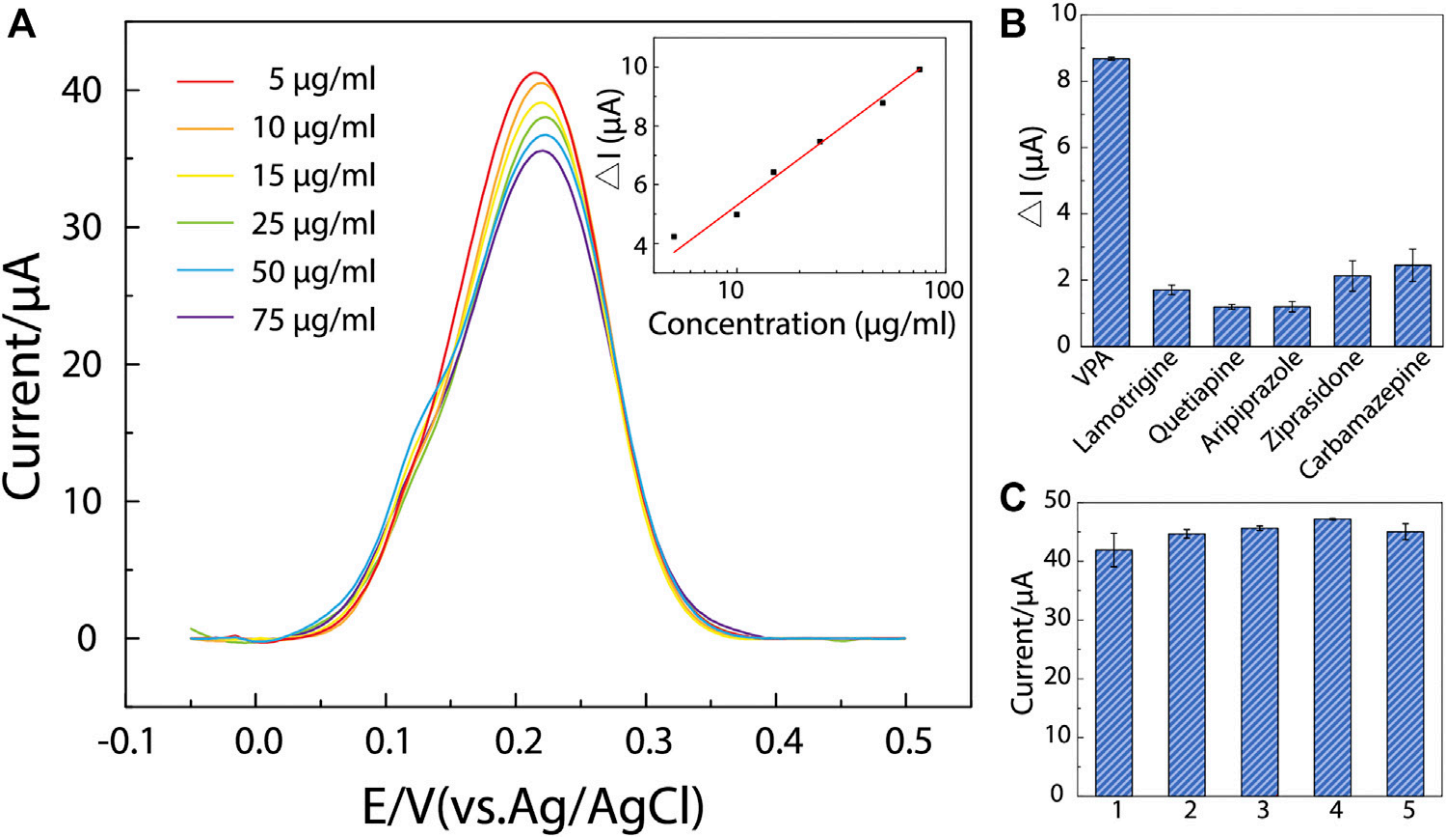
The determination performance, selectivity, and uniformity of ppy@AuNPs-MIP sensor. **(A)** DPV responses on ppy@AuNPs-MIP sensors incubated in VPA solutions whose concentrations ranged from 5 to 75 μg/ml (from top to bottom) for 8 min. The inset: linearity curve of ΔI and VPA concentration (*R* ^2^ = 0.99). **(B)** DPV responses of VPA and interferents (*n* = 3). **(C)** The designed sensors showed good uniformity (*n* = 3). Error bars got from triplicate measurements on each modified electrode.

In research detecting analytes based on the MIP/gate effect, a log-linear correlation is often expected between the analyte concentration and the change in peak current intensity (Liu et al., 2016; Liu et al., 2017; Parlak et al., 2018). In this work, prior to measurements, ppy@AuNPs-MIP-modified SPEs were incubated in a series of VPA solutions (concentrations ranged from 5 μg ml^−1^–75 μg ml^−1^) for 8 min under mild stirring. Figure 4A shows the DPV response of each SPE. With VPA concentration increasing, the peak current intensity decreased, which was consistent with the MIP/gate effect. Quantitatively, compared to that before incubation, the change in peak current intensity after each SPE was incubated in certain VPA solutions (ΔI) exhibited a logarithmic relevance with VPA concentration (C). The calibration equation is ΔI = 4.95 lgC + 0.50 (ΔI: μA; C: μg ml^−1^), with the correlation coefficient of 0.99 (Figure 4A, inset). And the estimated LOD obtained was down to 17.48 μM (2.91 μg ml^−1^) (S/N = 3). The saturation effect of recognition sites in MIP likely accounted for the observed logarithmic relevance. As VPA concentration increased, the limited number of recognition sites in MIP tended to be saturated, causing the sensor to be less sensitive to the concentration change in solutions. This suggested that optimization of MIP fabrication was essential for sensor improvement: a small number of recognition sites led to the saturation of MIP in low concentration, restricting the detection range to a narrow scale, while MIP with too many recognition sites lowered the sensitivity of the sensors.

Although the designed sensor behaved satisfactorily within the concentration of 5–75 μg ml^−1^, its response range failed to completely cover the VPA therapeutic window (50–100 μg ml^−1^) (Collins-Yoder and Lowell, 2017; Verrotti et al., 2020). Generally, when VPA is used to treat BD, its blood concentration needs to be above 50 μg ml^−1^. Our sensor is thus capable of indicating the drug’s effectiveness but unable to alert its adverse effects. To improve the sensor’s detection performance, further optimization of MIP fabrication to obtain an adequate number of recognition sites is required, especially a rigorous investigation of the adsorption characteristics of ppy@AuNPs-MIP and ppy@AuNPs-NIP to fully understand the characteristics of the MIP (Xue et al., 2014; Han et al., 2016).

### Selectivity and Uniformity Test

The selectivity of the designed sensor was evaluated under the interference of lamotrigine, quetiapine, aripiprazole, ziprasidone, and carbamazepine—drugs that were commonly used in clinical combination therapies with VPA for BD treatment (Geoffroy et al., 2012; Lindstrom et al., 2017; McIntyre et al., 2020). Similar to VPA determination, ppy@AuNPs-MIP-modified SPEs were incubated in different drug solutions (50 μg ml^−1^) mentioned above for 8 min, and then their DPV responses were recorded (Figure 4B). Obviously, the DPV responses of interferents were much lower than those of VPA. It is noteworthy that the actual therapeutic window of interferents is much lower than 50 μg ml^−1^ (lamotrigine: 3–14 μg ml^−1^, quetiapine: 0.1–0.5 μg ml^−1^, aripiprazole: 0.1–0.5 μg ml^−1^; ziprasidone: 0.05–0.2 μg ml^−1^; carbamazepine: 4–12 μg ml^−1^) (Mauri et al., 2018). The result demonstrated that our sensor had a good selectivity to VPA.

Moreover, as attributed to the highly controllable fabrication process, our sensor showed a good uniformity (Figure 4C), which was necessary for further mass production and potential clinical use.

## Conclusion

In this work, we constructed a novel MIP-electrochemical biosensor for VPA determination with high selectivity and sensitivity. The critical point of this strategy is to fabricate VPA-MIP with simple one-step electropolymerization and employ the MIP/gate effect to realize sensitive concentration–electrical signal response. Our work took a successful step to overcome difficulties in convenient control of drug concentrations for patients with bipolar disorder. Further studies will focus on the optimization of the MIP fabrication to enlarge the response range, tests in real samples, and integration with portable devices for clinical use.

## Data Availability

The original contributions presented in the study are included in the article/Supplementary Material; further inquiries can be directed to the corresponding authors.
